# Application of conventional molecular dynamics simulation in evaluating the stability of apomyoglobin in urea solution

**DOI:** 10.1038/srep44651

**Published:** 2017-03-16

**Authors:** Dawei Zhang, Raudah Lazim

**Affiliations:** 1 School of Physics and Engineering, Henan University of Science and Technology, Luoyang 471023, P. R. China; 2 Division of Chemistry and Biological Chemistry, School of Physical and Mathematical Sciences, Nanyang Technological University, Singapore 637371, Singapore

## Abstract

In this study, we had exploited the advancement in computer technology to determine the stability of four apomyoglobin variants namely wild type, E109A, E109G and G65A/G73A by conducting conventional molecular dynamics simulations in explicit urea solution. Variations in RMSD, native contacts and solvent accessible surface area of the apomyoglobin variants during the simulation were calculated to probe the effect of mutation on the overall conformation of the protein. Subsequently, the mechanism leading to the destabilization of the apoMb variants was studied through the calculation of correlation matrix, principal component analyses, hydrogen bond analyses and RMSF. The results obtained here correlate well with the study conducted by Baldwin and Luo which showed improved stability of apomyoglobin with E109A mutation and contrariwise for E109G and G65A/G73A mutation. These positive observations showcase the feasibility of exploiting MD simulation in determining protein stability prior to protein expression.

Over the last few decades, researches involving proteins have extended beyond the scope of solely exploring the relationship between proteins’ structure and function to one that involves altering the structures and functions of protein biomolecules for beneficial use in biotechnological innovations in both laboratory and industrial application^1^,^2^,^3^,^4^,^5^,^6^. Designing structurally and functionally stable proteins are crucial for industrial applications and laboratory-based researches as a slight deviation in surrounding condition from their original cellular environment may lead to the denaturation of proteins, which may destroy their biological functionality^1^,^2^,^3^,^4^,^5^,^6^. To accommodate to the highly sensitive nature of proteins, protein modifications need to be done such that the stability of the biomolecule is enhanced while still being biologically functional under conditions different from their native environment^1^,^2^,^3^,^4^,^5^,^6^. Improving the stability of proteins when exposed to extreme conditions is highly practiced in industries and research laboratories due to advantages such as faster chemical reactions, improved solubility of substrates, better immunity towards microbial contamination and easier storage and handling of proteins, all of which will boost the efficiency of both industrial processes and laboratory work^4^,^5^,^6^.

Altering the structures and functions of proteins in a bid to design and create proteins with superior applications and better stability compared to their natives are made possible through the inauguration of protein engineering^6^,^7^,^8^. The rise of this multidisciplinary technology saw the development of tools proficient in modifying proteins to augment stability, selectivity and catalytic activity of enzymatic proteins and at the same time allow for better efficiency in the design of novel drug molecules with desirable activities^6^,^7^,^8^. Mimicking nature, protein engineering utilizes mutagenesis and sequence variation to change the physical and chemical properties of proteins to achieve desired applications^1^,^6^,^7^,^8^. Mutations that stabilize the secondary structures of native proteins and mutations that enhance the intermolecular interactions within protein and between protein and ligand/solvent are some of the techniques used in research to improve the stability of proteins in solution^7^,^8^,^9^,^10^. However, determining the residue to be mutated is a complex task that requires thorough understanding of the three-dimensional structure of proteins as destabilization of the tertiary structure may occur upon mutation, which may lead to the loss of protein functions^7^,^8^.

Incorporation of modern computational tools in protein engineering had enabled the exploration and comprehension of protein dynamics, structures and functions at atomistic level^7^,^8^. Molecular dynamics (MD) simulation is one among a variety of contemporary computational devices that are commonly used in computational protein engineering, an area which is gaining tremendous interest as an affordable and effective alternative for rational or knowledge-based design of proteins^7^,^8^. Theoretical studies utilizing MD simulation empowered in-depth observation of the intricate dynamics of biological macromolecules thus permitting the understanding of protein folding and unfolding, protein stability and conformational changes^11^. These interesting applications offered by MD simulation are particularly useful in designing proteins as it allows one to provide insights on the feasibility of the mutation performed in maintaining the overall conformation of the native protein while simultaneously providing information regarding crucial interactions (e.g. hydrophobic interactions, Van der Waals, hydrogen bond) that may govern the stability of the studied protein. These aspects of MD simulation aid in eliminating the chances of disrupting the tertiary structure of the native protein when mutagenesis are conducted experimentally rendering MD simulation an attractive technology that can be exploited to increase the efficiency and ease the workload and expenditure of experimental researchers involved in protein research.

To verify the practicality of using MD simulation to predict protein stability, MD simulations for wild type apomyoglobin (apoMb) and three of its mutants namely E109A (Helix G), E109G (Helix G) and G65A/G73A (Helix E) were conducted in explicit urea solution, which acts as a denaturant triggering the unfolding of the apoMb variants. ApoMb is a small protein derived from myoglobin through the removal of heme^12^. At near neutral pH, apoMb is comprised of eight helical domains labeled A to H (Fig. 1) and its overall structure is similar to that of myoglobin except for a few features such as partial unfolding of some domains namely helix F, N-terminal end of Helix G and C-terminal end of Helix H^12^,^13^. These slight differences in structure between holomyoglobin and apoMb were addressed through NMR studies conducted by Eliezer and Wright who observed more fluctuations at loop EF, Helix F, loop FG and the beginning of Helix G for apoMb^13^. ApoMb is an ideal model for the folding studies of globular, single-domain proteins due to the accessibility of its intermediates whose stabilities stand in-between that of folded and unfolded states^14^,^15^,^16^,^17^,^18^,^19^. This in turn boosts the popularity of apoMb in the research arena rendering the information pertaining to apoMb easily accessible and this is highly preferred in computational studies where empirical evidence is greatly appreciated. From the trajectories obtained, various analyses aiding in disclosing the difference in stability among the apoMb variants will be conducted in this study. The stability pattern determined through this theoretical work will be compared with the stability ranking of apoMb variants determined by Luo *et al*. who studied the role of helix propensity on the stability of six apoMb mutants namely E109A, E109G, Q8A, Q8G, G23A/G25A and G65A/G73A using reversible urea unfolding observed through circular dichroism (CD) and fluorescence (FL)^9^.

## Methodology

Two MD simulations were conducted for each of the apoMb variants namely wild type, E109A, E109G and G65A/G73A using AMBER10 simulation package^20^. The X-ray crystal structure of wild type myoglobin from sperm whale was obtained from Protein Data Bank (PDB) with PDB id of 1BZP and was used as a template in the modeling of the wild type apoMb^21^. The heme moiety, crystallization waters and sulphate ions included in the X-ray structure were removed prior to any calculations. Before performing simulations of apoMb variants in explicit urea solution, the wild type apoMb was solvated in an octahedral TIP3P water box and relaxed for 500 ps at neutral pH after the heme was removed^22^. The steps for this simple equilibration are described in the supporting information (SI). The last frame of the trajectory obtained from the relaxation of wild type apoMb in water was acquired for manual mutation of residues to obtain the mutated proteins of interest. Upon mutation, hydrogen atoms were added and all histidine residues were doubly protonated to represent the state of apoMb at pH 4.2. These steps are simplified with the aid of the LEaP module in AmberTools 1.2^20^.

Since the simulation will be conducted in explicit urea solution, the urea system was constructed by diluting the pre-equilibrated 8 M urea box available in AMBER 10 simulation package using TIP3P water molecules to obtain a urea concentration of approximately 2 M, giving rise to an enclosed rectangular box of dimensions 69.58 Å by 66.60 Å by 72.52 Å which contained 330 urea molecules and 9118 water molecules^20^,^22^. Upon preparation of the urea solution, the entire system was minimized using steepest descent method for 10000 steps followed by another 10000 steps of minimization using conjugate gradient method. The urea system was heated from 10 K to 277.15 K in 100 ps using Langevin thermostat with a collision frequency of 4 ps^−1^ in canonical (NVT) ensemble^23^,^24^. After heating, a 3 ns simulation under isothermal-isobaric (NPT) ensemble was conducted for the urea system to ensure proper mixing of the urea-water mixture under constant temperature (277.15 K) and pressure. Generalized Amber force field (gaff) was employed to conduct the simulation outlined above^25^. Parameters and charges used for urea molecule were obtained from Özpınar *et al*.^26^.

Upon construction of the equilibrated 2 M urea box, the prepared wild type apoMb and interested mutants of apoMb namely E109A, E109G and G65A/G73A were solvated in the urea-water mixture enclosed in an octahedral box with the minimum distance between protein and edge of box set to 15 Å. Using the LEaP module, Na^+^ and Cl^−^ ions were added to balance the charge of the system^20^. Prior to the production run conducted to mimic the unfolding of the apoMb variants as done experimentally by Luo *et al*., the solvent molecules surrounding the protein was initially minimized using steepest descent method for 10000 steps followed by another 10000 steps of minimization using conjugate gradient method. The entire protein + solvent system was subsequently minimized for 5000 steps using steepest descent method and further minimized using conjugate gradient method until the energy gradient of the protei + solvent system converges to 0.01 kcal/mol/Å. Ensuing the minimization step, heating of the entire system from 10 K to 300 K using Langevin thermostat with a collision frequency of 4 ps^−1^ were conducted for 100 ps in NVT ensemble^23^,^24^. The protein was weakly restrained using a harmonic potential of 5 kcal/mol/Å^2^ during the heating process to prevent large fluctuations of the protein on temperature change. After heating the simulated system to 300 K, production run for each protein was conducted over a period of 20 ns using NPT ensemble. Two sets of 20 ns simulations were conducted for each apoMb variants. A time step of 2 fs was used for all the simulations conducted in this study with long-range electrostatic interactions calculated using particle mesh Ewald method and covalent bonds involving hydrogen atoms constrained using SHAKE algorithm^27^,^28^. AMBER ff99SB and gaff are the force fields used to conduct all simulations involving apoMb variants in explicit urea solution^25^,^29^,^30^.

## Results and Discussion

The stability of eight apoMb variants *viz.* wild type, E109A, E109G, Q8A, Q8G, K140A, G23A/G25A and G65A/G73A had been explored by Luo *et al*. experimentally through reversible urea unfolding of these proteins which were monitored through CD and FL^9^. Based on the difference in free energies calculated by Luo *et al*. for each mutant relative to wild type, E109A, Q8A, K140A and G23A/G25A were shown to have better stability than wild type while E109G and Q8G is less stable compared to wild type^9^. G65A/G73A mutation was the only mutation that was reported by Luo *et al*. to exert little effect on the stability of apoMb^9^. With the aim of reproducing the stability ranking of the apoMb variants revealed through the aforementioned study, we performed MD simulations for four selected apoMb variants namely wild type, E109A, E109G and G65A/G73A in explicit 2 M urea model to mimic the reversible urea unfolding experiments carried out by Luo *et al*.^9^. Trajectories acquired were analyzed to verify the stabilities of the four apoMb variants against the results obtained by Luo *et al*. by monitoring the extent of denaturation of the variants through observations related to protein dynamics and conformational changes that might occur during the simulations^9^. Since two simulations were conducted for each apoMb variants, data analyzed from the two simulations were averaged and presented in this paper.

### Root-mean-square deviation (RMSD)

Calculating the root-mean-square deviation (RMSD) of proteins permits the quantification of the degree of conformational changes that may occur during MD simulations. To examine the change in the conformation of myoglobin with the removal of the heme group and subsequent mutation to obtain E109A, E109G and G65A/G73A, RMSDs of the apoMb variants relative to the crystal structure of sperm whale myoglobin (PDB id: 1BZP) were calculated^21^. Since the most staggering difference between myoglobin and wild type apoMb as observed by Eliezer and Wright through NMR lies in Helix F and part of Helix G and H, the RMSD of Helix F to H of the apoMb variants (wild type, E109A, E109G and G65A/G73A) with respect to the crystal structure of myoglobin (1BZP) were calculated and plotted as shown in Fig. 2A 13,21. Based on the plots presented in Fig. 2A, E109G clearly showed the highest increase in RMSD during the last 10 ns of the simulation indicating significant changes in the conformation of apoMb at helix F to H in the presence of E109G mutation compared to others. The large variation in conformation may be one of the factors contributing to the destabilization of apoMb upon E109G mutation. This observation corroborated the destabilizing effect of E109G mutation reported by Luo *et al*.^9^.

Due to the proximity of the RMSD plots of wild type, E109A and G65A/G73A, conformational changes at helix F to H could not be compared through the RMSD versus time graph. Hence, probability distributions of RMSDs calculated over a period of 20 ns were plotted as shown in Fig. 2B to compare the extent of conformational change among the apoMb variants. The free energy differences of E109A, E109G and G65A/G73A relative to wild type apoMb were reported by Luo *et al*. to be 0.17, −0.89 and −0.11 kcal/mol respectively^9^. The RMSD distribution curve of E109A is located slightly to the left of the RMSD distribution curve of wild type apoMb (Fig. 2B). This suggests that E109A undergoes lesser conformational change compared to wild type. On the other hand, RMSD distribution curves of E109G and G65A/G73A are located on the right side of the plot for wild type indicating more changes in the structure of apoMb at helix F to H upon E109G and G65A/G73A mutations. While extent of conformational change is not a direct measure of the stability of the apoMb variants, the larger deviations in structures of E109G and G65/G73A compared to wild type concurred with the destabilizing effect of E109G and G65/G73A mutations on wild type apoMb. The next step is to explore the intermolecular interactions within the protein, which may provide us with better indications on the stability of the protein upon mutation.

### Native Contacts

Native contact quantifies the number of interactions between spatially closed amino acids, which are not sequentially next to each other in the primary sequence of the protein. The fraction of native contacts preserved reflects the stability of the proteins in the presence of denaturants. Proteins that are less stable will tend to unfold more rapidly leading to a greater loss in native contacts over the same period of simulation time. The variation of native contacts with time for all apoMb variants studied showed a descending trend that is in accordance to the low pH condition and the presence of urea denaturants. E109G mutation, which is suggested to have a destabilizing effect, exhibited the largest drop in native contacts as the simulation progresses (Fig. 3). On the contrary, E109A has the highest percentage of native contacts preserved hence showcasing the improved stability of apoMb with E109A mutation (Fig. 3). The native contacts preserved in G65A/G73A, on the other hand, is relatively similar to WT corroborating the none to slight destabilizing effect of this mutation as reported by Luo *et al*.^9^.

### Solvent Accessible Surface Area (SASA)

Other than the general consideration of interactions within the protein in terms of native contacts, a crucial intermolecular interaction within globular proteins that deserves attention is hydrophobic interaction. Hydrophobic interactions established among non-polar amino acids ensure the stability of globular proteins in solution by shielding the non-polar amino acids in hydrophobic cores, away from the aqueous environment^31^. In experimental studies, hydration of the hydrophobic core during protein unfolding and hydrophobic collapse during the start of protein folding are frequently monitored using UV fluorescence spectroscopy^9^,^12^,^32^,^33^,^34^,^35^,^36^,^37^. This spectroscopy technique exploits the intrinsic fluorescence property of proteins to provide sensitive indications of variation in the solvent accessibility of the hydrophobic core caused by changes in tertiary structure^32^,^33^,^34^,^35^. During denaturation, unfolding of proteins inevitably causes the hydrophobic core to be exposed to the aqueous surrounding leading to the loss of hydrophobic interactions among non-polar amino acid clusters. In the case of apoMb, information with regard to structural changes during folding and unfolding is acquired by monitoring the fluctuations in the fluorescence emission of Trp7 and Trp14, both of which are components of the hydrophobic core of apoMb which are confined by Helices A, G and H (Fig. 4A)^12^,^32^,^33^,^34^,^35^,^38^.

Theoretically, changes in the accessibility of protein to solvent can be determined by computing solvent accessible surface area (SASA). In this study, SASA of apoMb variants were acquired using the ptraj module in AmberTools^20^. The “surf” command in the ptraj module implemented the linear combination of pairwise overlaps (LCPO) algorithm by Weiser *et al*. to compute SASA of proteins^20^,^39^. During the course of the simulations conducted, the SASA of the apoMb variants will naturally get larger as hydration of the hydrophobic core occurs during unfolding causing the interruption of hydrophobic interactions among non-polar residues. The calculation of the SASA of the entire protein showed increasing trends for E109A, G65A/G73A, wild type and E109G indicating the solvation of the hydrophobic core as unfolding proceeds (Fig. 4B). From the graph of SASA versus time plotted in Fig. 4B, E109G showed a steeper increase while E109A showed a more gradual increase in SASA compared to wild type. The increase in SASA of the whole protein for the double mutation, on the other hand, are relatively similarly to that of wild type. These observations are in agreement with experimental observations whereby the stability of the apoMb was improved with E109A mutation while destabilization of the apoMb was observed for both E109G and G65A/G73A mutation, with the latter having none to slight destabilizing effect on apoMb^9^.

The order of the stability of the four apoMb variants were also reproduced through the plotting of the SASA of Trp7 versus simulation time shown in Fig. 4C which provide direct observation on the accessibility of the hydrophobic core to enclosing solvent since Trp7 is part of the hydrophobic core. As the simulations were adequately conducted to monitor early unfolding of apoMb, only the SASA of Trp7 was measured as this residue is closer to the protein surface and fluctuations in SASA acquired will be more substantial compared to Trp14 which will remain embedded in the hydrophobic core during the course of the simulation (Fig. 4A)^32^,^33^,^34^,^35^. The distribution curves related to the SASA of Trp7 for all four variants were also depicted in Fig. 4D to provide a clearer illustration of the accessibility of Trp7 to enclosing urea solution. The distribution curves of wild type and G65A/G73A peaked at approximately similar values indicating the minimal effect that the double mutation has on the stability of apoMb. Using the distribution curve for wild type as reference, Fig. 4D evidently showed that the hydrophobic core is more protected from the external environment upon E109A mutation as demonstrated by the smaller peak value compared to that of wild type. On the other hand, the hydrophobic core of apoMb becomes more accessible to the aqueous environment upon E109G mutation as suggested by the larger peak value of its distribution curve compared to wild type. These observations point toward the importance of hydrophobic core stabilization in ensuring the stability of apoMb as minimal exposure of the hydrophobic core to surrounding solvent, portrayed in E109A, led to the enhanced stability of E109A relative to wild type apoMb.

### Correlation Map

Other than comparing the stability of the apoMb proteins by analyzing their overall configuration (*vide supra*), some interest were also invested into looking at the dynamical differences during the unfolding of the four apoMb variants which may provide us with some insights pertaining to the dissimilarity in the stability of the proteins in 2 M urea solution. Calculation of correlation matrix is one method that is frequently utilized to depict dynamical information of proteins in two-dimensions^40^,^41^,^42^. To observe the correlation in the dynamics of the helical domains and loops of apoMb, correlation matrices over the last 5 ns of one of the trajectories acquired for each apoMb variants were plotted (Fig. 5A). The red regions in the correlation maps in Fig. 5A signify the concerted movement of residues in the same direction while the blue regions represent fluctuations, which are anti-correlated. Correlations between structural domains in apoMb may arise through specific contacts while others may be due to long-range interactions. Hence, equipped with the correlation maps plotted in Fig. 5A and the understanding of the three-dimensional configuration of apoMb, we are able to indirectly infer the presence of interactions between or among secondary structures of the apoMb variants.

From the correlation maps acquired, four regions with substantial differences in correlation intensities across the correlation maps of the four apoMb variants had been identified and labeled as shown in Fig. 5A. Region 1 represents the correlated motions of Helices A, G and H and loop GH that enclosed the hydrophobic core of apoMb, often termed the AGH-core (Fig. 5A and B)^12^,^14^,^34^,^38^,^43^,^44^. As one of the first feature established during the folding process of apoMb and under conditions of low pH and high temperatures, the AGH-core remained preserved through hydrophobic interactions albeit the partial unfolding of Helices A, G and H^12^,^14^,^34^,^38^,^43^,^44^. By comparing the dynamical correlation intensities of Helices A, G and H and loop GH, the stability of the AGH-core may be inferred since a positive correlation implies the concerted movement of residues in this domain which is only possible when hydrophobic interactions among residues in the hydrophobic core are conserved. In the presence of urea and under acidic conditions, the AGH-core may be slightly destabilize and this is most apparent in E109G whereby Helices A, G and H and loop GH showed the weakest correlation in motion compared to the other proteins. On the contrary, E109A displayed better correlation in motion of residues in the AGH-core compared to wild type hence indicating enhanced stability of the AGH-core of E109A compared to wild type. For the double mutation, the AGH-core showed similar correlation in motion as E109A thus showcasing the improved stability of the AGH-core of G65A/G73A compared to wild type. However, according to Luo *et al*. G65A/G73A mutation led to the slight disruption of apoMb stability and this fact had been corroborated in this study through analyses discussed earlier^9^. The enhanced stability of the AGH-core in G65A/G73A might suggest the possibility of other structural features contributing to the destabilization of G65A/G73A besides the AGH-core.

Solely using the stability of the AGH-core as a descriptor for the overall stability of apoMb is an overstatement as other structural features may play an instrumental role in maintaining the stability of apoMb as well. Regions 2 and 3 labeled in Fig. 5A correspond to correlation in dynamics between Helix A and loop EF and between Helix H and loop EF respectively (Fig. 5C). E109G and G65A/G73A showed weaker correlation in the direction of motion of domains involved in Region 2 and 3 relative to wild type while the dynamical correlation of these domains were similar for both wild type apoMb and E109A. The lower correlation in the direction of motion between Helix A and loop EF and Helix H and loop EF in E109G and G65A/G73A may have ensued due to the diminishing interaction between Helix A, Helix H and loop EF which may come in the form of hydrogen bond breaking which will be discussed in a later section of this paper. The loss of these interactions caused Helix A and Helix H to move away from loop EF making the hydrophobic core more accessible to surrounding solvent. In addition, by comparing the correlation intensities of Region 1 to 3 in Fig. 5A across the four apoMb variants, the significant role of hydrophobic core stabilization toward the stability of apoMb in solution is further emphasized and this is best portrayed by E109A which exhibits better stabilization of the hydrophobic core which is characterized by the greater correlation in motion of residues in the AGH-core, Helix A, Helix H and loop EF compared to other apoMb variants (Fig. 5A).

**Figure 1:**
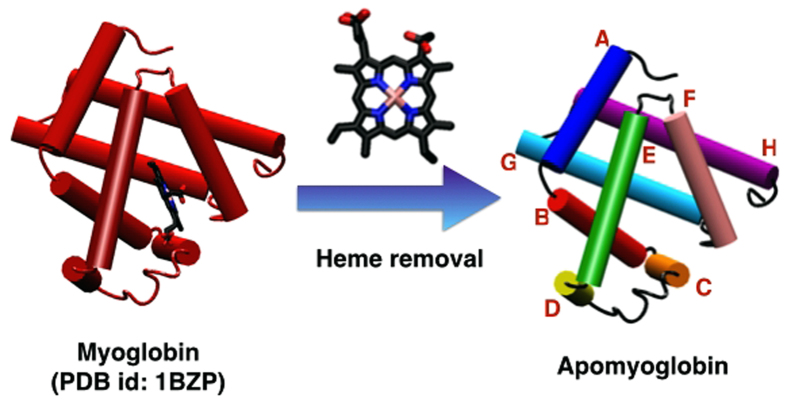
Cartoon representation of myoglobin and apomyoglobin with the individual helices alphabetically labeled A to H.

**Figure 2:**
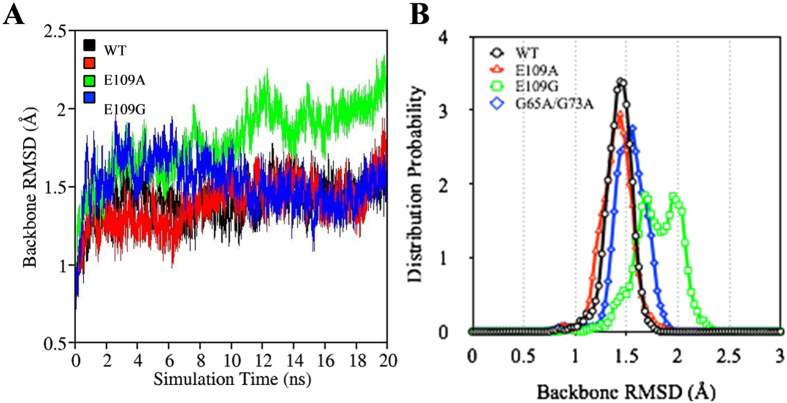
(**A**) Variation in RMSD of WT (black), E109A (red), E109G (green) and G65A/G73A (blue) with time for Helix F to H. (**B**) RMSD distribution of WT (black), E109A (red), E109G (green) and G65A/G73A (blue) for Helix F to H.

**Figure 3:**
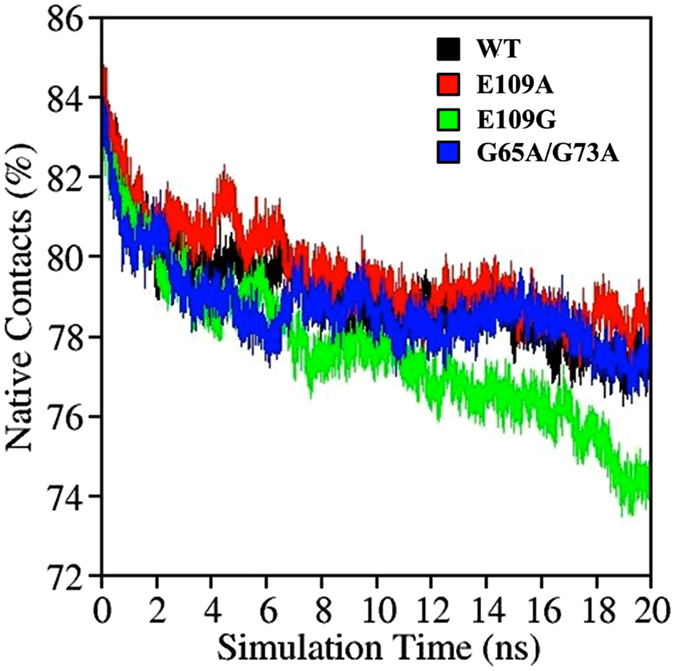
Variation in native contacts with time for WT (black), E109A (red), E109G (green) and G65A/G73A (green).

Another region of interest that will be explored in this section is the structural domains of apoMb that surrounds the heme pocket that is left vacant after the removal of heme from myoglobin to produce apoMb (Fig. 1). A coarse-grained dynamic Monte Carlo simulation conducted by Haliloglu and Bahar observed large fluctuations in segments of apoMb namely Helix F, loop FG, and sections of apoMb starting from the C-terminus of the Helix B to the N-terminus of Helix E, all of which are located in the vicinity of the heme pocket (Fig. 5D)^45^. These intensified fluctuations were attributed to the tendency of the segments highlighted in Fig. 5D to collapse into the vacant space which was previously occupied by heme^45^. The propensity of these domains to collapse into the empty heme pocket was reproduced in the simulations conducted here as positive correlations were observed in Region 4 of the correlation maps of all apoMb variants which suggests the probable development of contacts among Helix F, loop FG, and sections of apoMb starting from the C-terminus of the Helix B to the N-terminus of Helix E as these domains moved into the vacant heme pocket^44^. Comparing the correlation intensities across the four apoMb variants, E109G portrayed the weakest correlated dynamics in Region 4 while the rest of the apoMb variants showed similar correlation intensities. The lack of correlation in the motion of the domains highlighted in Fig. 5D for E109G hinted toward the loss of contact among these domains, which might lead to the greater accessibility of the heme pocket to solvent molecules. This might expedites the denaturation process of E109G relative to other apoMb variants by encouraging the solvation of the hydrophobic core, which is adjacent to the heme-binding pocket.

### Principal Component Analysis (PCA) and RMSF

Under denaturing conditions, several events related to the unfolding of apoMb could be monitored through short MD simulations which include elevated fluctuations in loops and helices specifically loop EF and Helix F and the loosening of the tightly packed hydrophobic core of apoMb^13^,^46^. This section will focus on exploiting the fluctuations in loop EF and Helix F to further corroborate the analyses conducted thus far which associate changes in overall conformation to the stability of the apoMb mutants relative to wild type. Principal component analysis (PCA) and root-mean-square fluctuations (RMSF) were computed to offer both visual and numerical observations of the degree of fluctuations occurring in loop EF and Helix F during the unfolding process of apoMb (Fig. 6). PCA is a well-established technique, which is useful for the study of protein dynamics as it limits the *3 N* (N = number of atoms in the protein) degrees of freedom of a protein to key degrees of freedom describing functionally crucial motions of the protein^47^,^48^. By performing PCA on one of the two trajectories obtained for each of the apoMb variants, crucial motion modes pertaining to the unfolding of apoMb could be identified from the trajectories and visualized using Interactive Essential Dynamics (IED) program with Visual Molecular Dynamics (VMD) as a display interface to observe and compare motion modes associated to fluctuations of loop EF and Helix F^49^,^50^. Greater fluctuations of loop EF and Helix F in comparison to wild type apoMb are expected for variants with destabilizing mutations and this was observed for E109G which showed the greatest fluctuations at loop EF and Helix F as seen in Fig. 6A. This observation was verified through the RMSF plots of residues within loop EF and Helix F as shown in Fig. 6B and C respectively.

**Figure 4:**
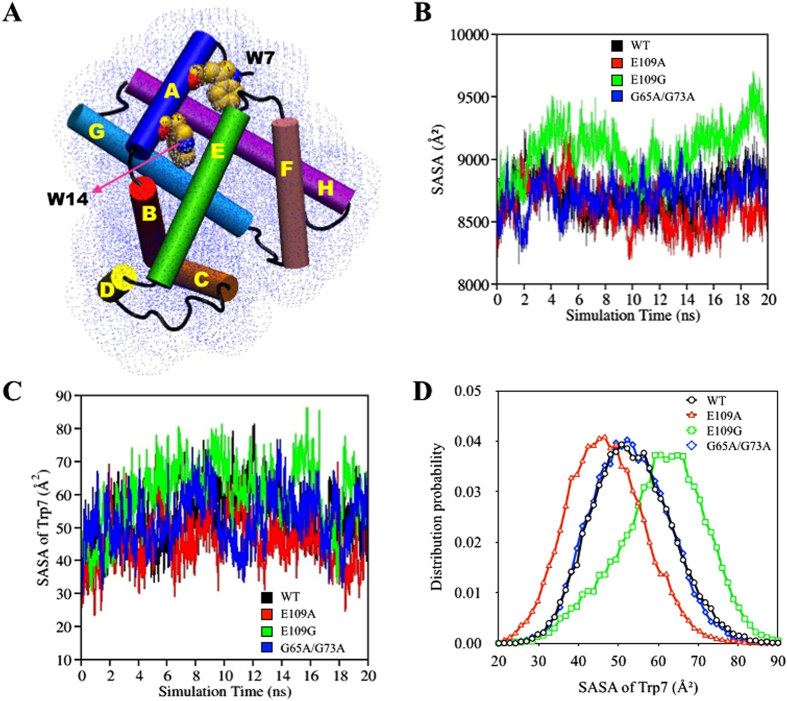
(**A**) Schematic representation of the solvent accessible surface area (SASA) of ApoMb with the helical domains and two residues found within the hydrophobic core namely Trp7 and Trp14 labeled. (**B**) Changes in the SASA of wild type (black), E109A (red), E109G (green) and G65A/G73A (blue) with time. (**C**) Changes in SASA with time for Trp 7 in wild type (black), E109A (red), E109G (green) and G65A/G73A (blue). (**D**) Distribution of SASA of Trp 7 for wild type (black), E109A (red), E109G (green) and G65A/G73A (blue).

**Figure 5:**
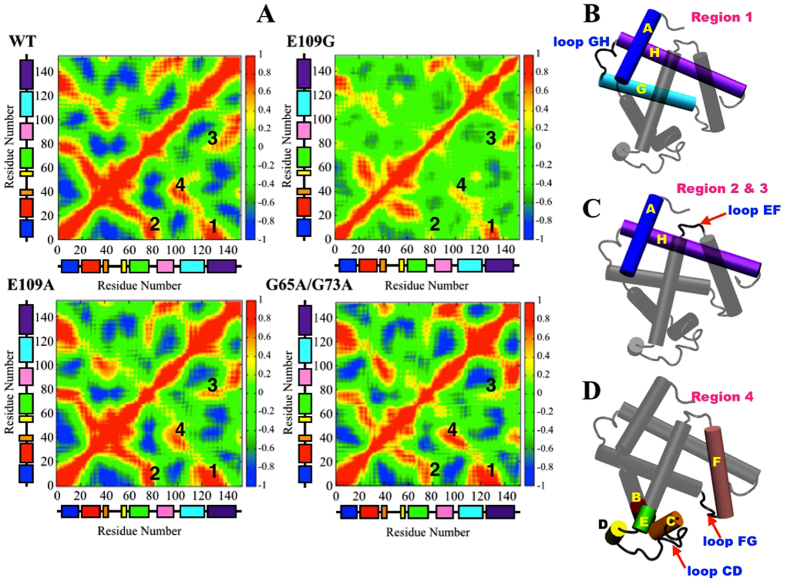
(**A**) Correlation map of wild type, E109A, E109G and G65A/G73A with the eight helical domains represented by color coded boxes going from Helix A (blue) to Helix H (purple). Schematic representation of apoMb with the helices and loops involved in (**B**) Region 1, (**C**) Region 2 and 3 and (**D**) Region 4 highlighted.

**Figure 6:**
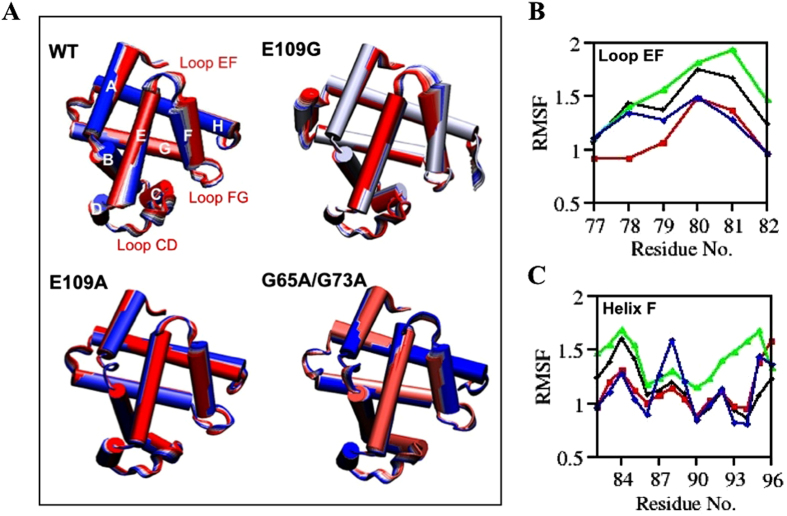
(**A**) Cartoon representations of fluctuations observed for WT, E109A, E109G and G65A/G73A obtained through PCA. The color transition from first to last frame goes from red to white to blue. Fluctuation of CA atoms with time for (**B**) Loop EF and (**C**) Helix F.

From the correlation maps of E109G in Fig. 5A, E109G showed the weakest correlation for motions corresponding to Helix F, loop FG, and sections of apoMb starting from the C-terminus of the Helix B to the N-terminus of Helix E (Fig. 5D). This observation may arise due to the larger fluctuations of loop EF and Helix F in E109G compared to other apoMb variants (Fig. 6B and C). The heightened fluctuations of loop EF and Helix F in E109G may cause Helix F and loop FG to move away from the vacant heme site leading to possible loss of contacts among the domains highlighted in Fig. 5D thus reducing the correlation in motions of these domains. In addition, the outward movement of loop EF and Helix F in E109G away from the empty heme pocket, as portrayed in Fig. 6A, also render the unoccupied heme site more accessible to surrounding solvent which may subsequently contribute to the disruption of the hydrophobic core of apoMb as demonstrated by the drastic increase in SASA of E109G compared to the other apoMb variants (Fig. 4B).

### Hydrophobic Core and Hydrogen Bonding

As protein folding progresses, a tightly packed hydrophobic core develops and this structural feature of globular proteins has been demonstrated by Munson *et al*. to be a critical physical characteristic which determines the stability, native structure and properties of proteins in general^51^. This information corroborated the analyses conducted hitherto in this study, which pointed toward the crucial role of hydrophobic core stabilization in enhancing the stability of apoMb that is best exampled by E109A. Hence, the next crucial step in this study is to understand the factors that contribute to the stability of the hydrophobic core, as this will enable us to acquire more information pertaining to the factors influencing the stability of apoMb, which is still lacking in detail. Since hydrophobic core is formed with the purpose of protecting the non-polar residues in the protein interior from the aqueous solution while having the polar residues lining the protein surface, we explored the role of hydrogen bonds established between side chains of polar residues on the circumference of apoMb in protecting the hydrophobic core from the aqueous environment. Using the ptraj module in AmberTools, hydrogen bond analysis were conducted for E109G to identify hydrogen bonds that form on the surface of the protein and at the same time may potentially serve as a gate allowing for the penetration of water into the hydrophobic core when the hydrogen bonds are broken during the unfolding process^20^. Since E109G showed the greatest increase in SASA relative to other apoMb variants (Fig. 5B), hydrogen bond analysis was only conducted for this mutant, as it will furnish us with a much clearer correlation between hydrogen bond disruption and the swelling of the hydrophobic core.

**Figure 7:**
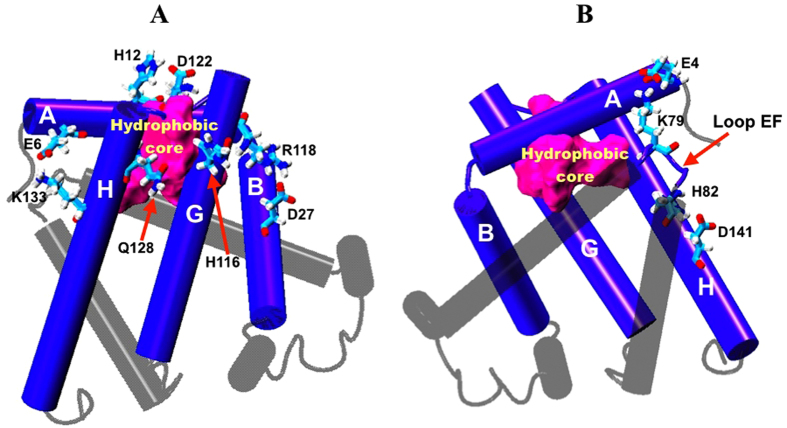
(**A**) Cartoon representation of apoMb with four hydrogen bonds between Glu6 and Lys133, His12 and Asp122, His116 and Gln128 and Asp27 and Arg118 displayed using licorice representation. (**B**) Cartoon representation of apoMb with two hydrogen bonds between Glu4 and Lys79 and His82 and Asp141 displayed using licorice representation. The hydrophobic core of apoMb is represented using surface representation coloured magenta.

**Figure 8:**
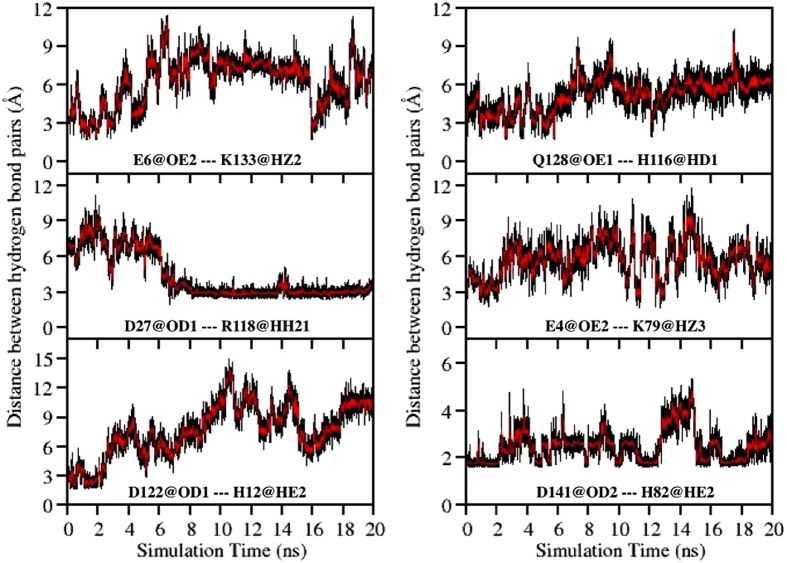
Variation in the distance between six hydrogen bond pairs namely Glu6@OE2—Lys133@HZ2, Asp27@OD1 ---Arg118@HH21, His12@HE2---Asp122@OD1, Q128@OE1---H116@HD1, E4@OE2---K79@HZ3 and D141@OD2---H82@HE2 as simulation progresses.

Six hydrogen bonds formed between (1) Glu6 (Helix A) and Lys133 (Helix H), (2) Asp27 (Helix B) and Arg118 (Helix G), (3) His12 (Helix A) and Asp122 (loop GH), (4) His116 (Helix G) and Gln128 (Helix H), (5) Glu4 (Helix A) and Lys79 (loop EF) and (6) His82 (loop EF) and Asp141 (Helix H), were identified from the hydrogen bond analysis conducted for E109G and exhibited in Fig. 7A and B. These hydrogen bonds are well distributed around the AGH-hydrophobic core and based on their location, the stability of these hydrogen bonds may influence the stability of the AGH-core as the disruption of these hydrogen bonds possibly expose the hydrophobic core to surrounding solvent. To apprehend the role of these hydrogen bonds in protecting the hydrophobic core from the external aqueous environment, the variations in the distances between the hydrogen bond pairs listed above were calculated and plotted in Fig. 8. With the exception of the newly formed hydrogen bond between Asp27 and Arg118, the rest of the hydrogen bonds identified (*vide supra*) showed signs of disruption as indicated by the increase in the distance between the hydrogen bond pairs as the simulation progresses (Fig. 8). The distance between the hydrogen bond pairs of His12 and Asp122, and Glu4 and Lys79 demonstrated a significant increase in length 2 ns into the simulation implying the breaking of these hydrogen bonds. This event was subsequently ensued by the breaking of two additional hydrogen bonds between Glu6 and Lys133 and between His116 and Gln128 about 2 ns later (Fig. 8). While obvious increases in distance between the four aforementioned hydrogen bond pairs were noted in Fig. 8, only constant fluctuations in the distance between His82 and Asp141 was observed suggesting the instability of the hydrogen bond formed between this residue pair.

Hydrogen bonds formed between Glu4 and Lys79, His12 and Asp122, and His82 and Asp141, may play a crucial role in shielding the hydrophobic core from the initial penetration of solvent molecules as disruption of these hydrogen bonds at the 2 ns mark (Fig. 8) led to a concomitant increase in the SASA of E109G at around the same simulation time (Fig. 4B). After 2 ns of simulation time, the SASA of E109G continued to increase and this may occur as a consequence of the disruptions of two additional hydrogen bonds between Glu6 and Lys133 and between His116 and Gln128 as the simulation advances. Further scrutinization of the distance plot in Fig. 8 also showed that the breaking of the hydrogen bond between His116 and Gln128 that began 5 ns into the simulation consequently led to the formation of a new hydrogen bond between Asp27 and Arg118 at the 6 ns mark leading to the separation of Helix G and H. This occurrence sequentially caused the hydrophobic core to be more susceptible to hydration from the external solution. Additionally, disruptions in the hydrogen bonds formed between His82 and Asp141 and His116 and Gln128 might also contribute to the almost non-existent correlation in motion between Helix A and loop EF and between Helix H and loop EF displayed in the correlation maps of E109G in Fig. 5A (Region 2 and 3 respectively). The disruption of these two hydrogen bonds might also account for the intensified fluctuation of loop EF and Helix F observed through PCA and RMSF plot in Fig. 6, which subsequently caused the domains highlighted in Fig. 5D to be poorly correlated in motion compared to wild type, E109A and G65A/G73A (Fig. 5A). These series of events eventually led to the hydration of the protein interior which in turn caused the loosening of the tightly packed hydrophobic core thus corroborating the diminished correlation among residues in the AGH-core exhibited in the correlation map of E109G in Fig. 5A (Region 1).

## Conclusion

In this work, we had conducted eight MD simulations of four apoMb variants namely wild type, E109A, E109G and G65A/G73A in explicit 2 M urea solution at pH 4.2. Trajectories obtained from the simulations conducted were analyzed for variations in RMSD, native contacts and SASA to compare the stability of the four variants theoretically which showed consistency with the stability determined experimentally by Luo *et al*. whom calculated the free energy difference of E109A, E109G and G65A/G73A relative to wild type apoMb to be 0.17, −0.89 and −0.11 kcal/mol respectively^9^. This showcased the feasibility of exploiting MD simulation for the determination of protein stability prior to protein expression.

Other than comparing the stability of apoMb variants, this study also investigated the factors that may contribute to the stability of apoMb. Combining the information acquired through the analyses of correlation maps, PCA and RMSF plots, hydrophobic core stabilization is revealed to be one of the main contributing factors in enhancing the stability of apoMb. In addition, we also investigated the role of hydrogen bonds in protecting the hydrophobic core from the external aqueous environment in order to comprehend in greater detail the features of apoMb that influence its stability. From the distance plots of hydrogen bond pairs identified through the hydrogen bond analysis conducted for E109G, the stability of the hydrophobic core was shown to be dependent on the stability of five hydrogen bonds formed between Glu6 and Lys133, His12 and Asp122, His116 and Gln128, Glu4 and Lys79 and His82 and Asp141 (Fig. 7). Disruption of these hydrogen bonds during the simulation led to the hydration of the hydrophobic core which is verified by the concomitant increase in SASA of E109G in Fig. 5B and C as the distance between the aforementioned hydrogen bond pairs began to increase. The ability of the MD simulation to provide information pertaining to factors influencing the stability of apoMb further emphasizes the practicality of utilizing MD simulation in protein research in a bid to lessen the effort required to determine mutations which have stabilizing effect on a protein of interest.

## Additional Information

**How to cite this article**: Zhang, D. and Lazim, R. Application of conventional molecular dynamics simulation in evaluating the stability of apomyoglobin in urea solution. *Sci. Rep.* **7**, 44651; doi: 10.1038/srep44651 (2017).

**Publisher's note:** Springer Nature remains neutral with regard to jurisdictional claims in published maps and institutional affiliations.
